# Pseudo-Yang-Lee Edge Singularity Critical Behavior in a Non-Hermitian Ising Model

**DOI:** 10.3390/e22070780

**Published:** 2020-07-17

**Authors:** Liang-Jun Zhai, Guang-Yao Huang, Huai-Yu Wang

**Affiliations:** 1 The School of Mathematics and Physics, Jiangsu University of Technology, Changzhou 213001, China; zhailiangjun@jsut.edu.cn; 2 Institute for Quantum Information & State Key Laboratory of High Performance Computing, College of Computer, National University of Defense Technology, Changsha 410073, China; guangyaohuang@quanta.org.cn; 3 Department of Physics, Tsinghua University, Beijing 100084, China

**Keywords:** Pseudo-Yang-Lee edge singularity, non-equilibrium phase transition, Kibble-Zurek scaling mechanism, non-Hermitian Ising model

## Abstract

The quantum phase transition of a one-dimensional transverse field Ising model in an imaginary longitudinal field is studied. A new order parameter *M* is introduced to describe the critical behaviors in the Yang-Lee edge singularity (YLES). The *M* does not diverge at the YLES point, a behavior different from other usual parameters. We term this unusual critical behavior around YLES as the pseudo-YLES. To investigate the static and driven dynamics of *M*, the (1+1) dimensional ferromagnetic-paramagnetic phase transition ((1+1) D FPPT) critical region, (0+1) D YLES critical region and the (1+1) D YLES critical region of the model are selected. Our numerical study shows that the (1+1) D FPPT scaling theory, the (0+1) D YLES scaling theory and (1+1) D YLES scaling theory are applicable to describe the critical behaviors of *M*, demonstrating that *M* could be a good indicator to detect the phase transition around YLES. Since *M* has finite value around YLES, it is expected that *M* could be quantitatively measured in experiments.

## 1. Introduction

As one of the most interesting phenomena in many body systems, quantum phase transition occurs when parameters of a system change through a critical point, and the occurrence indicates the emergence of new physics and new states of the system [1,2]. In most of the studies of quantum phase transition, the Hamiltonians are usually assumed as Hermitian ones. Nevertheless, owing to recent experimental progress [3,4,5,6,7,8,9], the phase transition in non-Hermitian systems has been drawing a great deal of interest, since they can present rich behaviors different from those of Hermitian systems [10,11,12,13,14,15,16,17]. Interesting characters have been found in various non-Hermitian systems, such as the parity-time (PT) symmetry breaking phase transition induced by interface state [18,19] and the real-complex energy spectrum transition accompanied by a many-body localization-delocalization phase transition [20]. Besides these studies of equilibrium phase transition, the non-equilibrium phenomena in non-Hermitian systems have also been studied [21,22]. It was found that the Kibble-Zurek scaling (KZS) mechanism was still applicable in describing the driven dynamics across the exceptional points (EPs), where the complex spectrum becomes gapless [21].

On the other hand, Yang and Lee were the first to connect phase transitions in non-Hermitian systems with zeros of the partition function, termed Lee-Yang zeros, in the complex plane of magnetic field [23]. They showed that the zero distribution approached the positive real axis and gave the transition point. When the applied symmetry-breaking field was imaginary, the edges of Lee-Yang zeros were singularity points in the thermodynamic limit [24]. These singularities were termed Yang-Lee edge singularities (YLES), and could be considered as a second order phase transition point with appropriate scaling laws [25,26,27,28,29,30]. The Lee-Yang zeros and YLES have been detected in recent experiments [31,32,33]. Theoretical investigations demonstrated that there were exotic scaling behaviors in the YLES, such as the negative correlation-length exponents, and divergence of order parameters [25]. The KZS mechanism has also been demonstrated to be applicable in the driven dynamics of YLES [34,35].

As one of the key features of YLES, the divergence of order parameter *P* depending on an applied field *h* usually exhibits as $P=|h-h_{\mathrm{YL}}^{L}{|}^{\sigma }$ with σ<0, where $h_{\mathrm{YL}}^{L}$ is the YLES point. For a one dimension (1 D) spin−1/2 Ising chain, several theoretical studies confirmed that σ=−1/2 [25,30,36,37,38,39]. By fine tuning the coupling strengths in the model, an unusual exponent of σ=−3/2 was found for a special three-state Potts model [40] and the 1 D spin-1/2 axial-next-to-nearest-neighbor model [41]. More recently, the exponent of σ=−1/2 has also been found in the scaling behaviors of the non-Hermitian Berry phase in YLES [42].

In the present research, we are going to define a new order parameter, which is not diverged at the YLES point, to character the critical behaviors around the YLES for the non-Hermitian Ising model. This order parameter obeys the similar scaling behavior with the usual order parameter but the critical exponent σ is positive. We termed this unusual scaling behavior around the YLES point as pseudo-YLES. The static behavior and the driven dynamics of this order parameter are studied in different critical regions, and KZS mechanism is still applicable to describing the behavior of this order parameter. Since the YLES points can appear at finite system size [34], the usual defined order parameter diverges at the YLES points even at small lattice size. However, the new defined order parameter is finite around the YLES points, and it is expected that this order parameter may be detected quantitatively in the experimental study.

This paper is organized as follows. In Section 2, the non-Hermitian 1 D Ising model subject to a transverse field and an imaginary longitudinal field is introduced, and the order parameter reflecting the pseudo-YLES is defined. In Section 3, the static behavior and driven dynamics of the order parameter are studied in different critical regions. A brief summary is given in Section 4.

## 2. The Non-Hermitian Ising Model and Order Parameter

We study a 1 D transverse-field Ising model with an imaginary longitudinal field, since this model is a typical and universal prototype to investigate the scaling behaviors in YLES [27,43,44]. The corresponding non-Hermitian Hamiltonian for a chain with length *L* is given by

(1) $$\begin{array}{c} H=-\sum_{n=1}^{L}\sigma_{n}^{z}\sigma_{n+1}^{z}-\lambda \sum_{n=1}^{L}\sigma_{n}^{x}-ih\sum_{n=1}^{L}\sigma_{n}^{z}, \end{array}$$

where $\sigma_{n}^{x}$ and $\sigma_{n}^{z}$ are the Pauli matrices at *n* site in the *x* and *z* direction, respectively. The second and third terms in Equation (1) respectively present a transverse real external field and a longitudinal imaginary field. When the imaginary field is absent, the Hamiltonian becomes Hermitian.

For the Hermitian Ising model, in order to research the classical phase transition, the ordinary order parameter is usually defined as

(2) $$\begin{array}{ccc} M_{C} & = & \mathrm{Tr}(Se^{-\beta H}/\mathrm{Tr}\left(e^{-\beta H}\right))\\ & = & \mathrm{Tr}\left[e^{-\beta H/2}Se^{-\beta H/2}\right]/\mathrm{Tr}\left(e^{-\beta H}\right), \end{array}$$

with S=±1 and β=1/T being the inverse temperature. $M_{C}$ is the ordinary magnetization. For the Hermitian system, $M_{C}$ is real and bounded in the range of [−1,1]. For the classical YLES, the order parameter can be defined in the same way of $M_{C}$, but the Hamiltonian is non-Hermitian. Since the applied magnetic field is complex, $M_{C}$ in YLES is usually complex and not bounded in the range of [−1,1]. Several analytic and numerical studies have demonstrated that $M_{C}$ diverges as $M_{C}\propto {\left(h-h_{\mathrm{YL}}^{L}\right)}^{-1/2}$ for the model of Equation (1) [25,34,35].

For the quantum YLES, the order parameter can be defined by a quantum-classical mapping. Since the *d*-dimensional quantum system can be mapped to a (d+1)-dimensional classical system, the order parament of the quantum YLES system can be defined as [27,34]

(3) $$\begin{array}{ccc} M_{Q} & = & \lim_{\beta \to \infty }\mathrm{Tr}\left[e^{-\beta H/2}\hat{M}e^{-\beta H/2}\right]/\mathrm{Tr}\left(e^{-\beta H}\right)\\ & = & \langle \psi_{g}^{L}|\hat{M}|\psi_{g}^{R}\rangle /\langle \psi_{g}^{L}|\psi_{g}^{R}\rangle , \end{array}$$

in which the operator $\hat{M}\equiv \sum_{n}^{L}\sigma_{n}^{z}/L$. $\langle \psi_{g}^{L}|$ and $\psi_{g}^{R}\rangle$ are the normalized left and right eigenvectors satisfying $H|\psi_{n}^{R}\rangle =E_{n}|\psi_{n}^{R}\rangle$ and $\langle \psi_{n}^{L}|H=\langle \psi_{n}^{L}|E_{n}$. Moreover, the eigenvectors usually form a biorthonormal basis

(4) $$\begin{array}{c} \langle \psi_{m}^{L}|\psi_{n}^{R}\rangle =\delta_{mn},\sum_{n}|\psi_{n}^{R}\rangle \langle \psi_{m}^{L}|=1. \end{array}$$

In the non-Hermitian model, the $M_{Q}$ is complex. It has been demonstrated that both the real and imaginary parts of $M_{Q}$, MR=Re(MQ) and MI=Im(MQ), could be used to describe critical behavior of YLES [34,35]. Around the YLES points, the static $M_{R}$ and $M_{I}$ scale as $M_{R}\propto {\left|h-h_{\mathrm{YL}}^{L}\right|}^{\sigma }$ and $M_{I}\propto {\left|h-h_{\mathrm{YL}}^{L}\right|}^{\sigma }$. For the (0+1) D YLES, σ=−1/2. Therefore, it should be difficult to quantitatively measure $M_{R}$ and $M_{I}$ in the experiment.

We think that it may be feasible to define some parameters that are possible to detect quantitatively experimentally. Here, we propose an order parameter evaluated in the space of the right eigenvector of the non-Hermitian system, as

(5) $$\begin{array}{ccc} M & = & \lim_{\beta \to \infty }\mathrm{Tr}\left[e^{-\beta H^{+}/2}\hat{M}e^{-\beta H/2}\right]/\mathrm{Tr}\left(e^{-\beta (H^{+}+H)/2}\right)\\ & = & \langle \psi_{g}^{R^{*}}|\hat{M}|\psi_{g}^{R}\rangle /\langle \psi_{g}^{R^{*}}|\psi_{g}^{R}\rangle , \end{array}$$

where $\langle \psi_{g}^{R^{*}}|$ means the Hermitian conjugation of the right eigenvector $|\psi_{g}^{R}\rangle$. It should be noted that $\langle \psi_{m}^{R^{*}}|$ and $|\psi_{n}^{R}\rangle$ are generally not orthonormal, $\langle \psi_{m}^{R^{*}}|\psi_{n}^{R}\rangle \neq \delta_{mn}$. When the Hamiltonian is Hermitian, this order parameter automatically goes back to what it should be. Since the operator $\hat{M}$ itself is Hermitian, the order parameter defined in Equation (5) is always real. Similar definition of order parameters can also be found in Refs. [20,21]. It will be shown below that *M* is not divergence at YLES critical points, which differs from the behaviors of usual order parameters of YLES. Recently, a method of computing mean values of observable by introducing a metric operator has been proposed [45,46], and the order parameter *M* can also be defined by introducing an adequate metric operator.

## 3. Pseudo-YLES Critical Behaviors

Before presenting our numerical results, we briefly introduce the phase structure of the model Equation (1). Since the imaginary longitudinal field has the same dimension as the real longitudinal field, Equation (1) has a ordinary ferromagnetic-paramagnetic phase transition (FPPT) point at $\left(g_{c},h_{c}\right)=\left(0,0\right)$ in thermal limit, where $g\equiv \lambda -\lambda_{c}$ and $\lambda_{c}=1$ [1]. Besides this FPPT phase transition, there are also critical points for YLES at $\left(g_{\mathrm{YL}}^{L},h_{\mathrm{YL}}^{L}\right)$ for g>0. The appearance of the YLES corresponds to the disappearance of energy gap between the ground state and the first excited state, which can induce critical phenomena. However, different from the FPPT, YLES can appear even at finite size, and the YLES critical points varies with system size *L*. Therefore, different critical regions coexist for this model: (i) around the FPPT critical point, the critical region belongs to the (1+1) D FPPT universe class; (ii) the (0+1) D YLES critical region appears near $h_{\mathrm{YL}}^{L}$ for a fixed *g*, and this critical region shrinks with the increase of *L*; (iii) the (1+1) D YLES critical region appears near $h_{\mathrm{YL}}^{\infty }$ for a fixed *g* and large *L*, and $h_{\mathrm{YL}}^{\infty }$ is the critical point of the infinite-size YLES. Since the (0+1) D YLES critical region appears at finite size, different critical regions can overlap with each other. For example, at large lattice size and small *g* the (0+1) D YLES critical region and (1+1) D FPPT critical region overlaps with each other. On the other hand, the (0+1) D YLES critical region overlaps with the (1+1) D YLES critical region near the $h_{\mathrm{YL}}^{\infty }$ for large lattice size. In these critical regions, it was shown that KZS is still applicable, and different scaling theories could be applicable simultaneously in the overlapping region [34,35].

In present study, to investigate the pseudo-YLES critical behaviors, the static and driven dynamic behaviors of *M* in the (0+1) D YLES, (1+1) D FPPT and (1+1) D YLES critical regions are numerically calculated. For the sake of clarity, all usual critical exponents of the three critical regions are listed in Table 1 [1,34,35].

### 3.1. Pseudo-YLES Critical Behaviors in the (0+1) D YLES Critical Region

**Figure 1:**
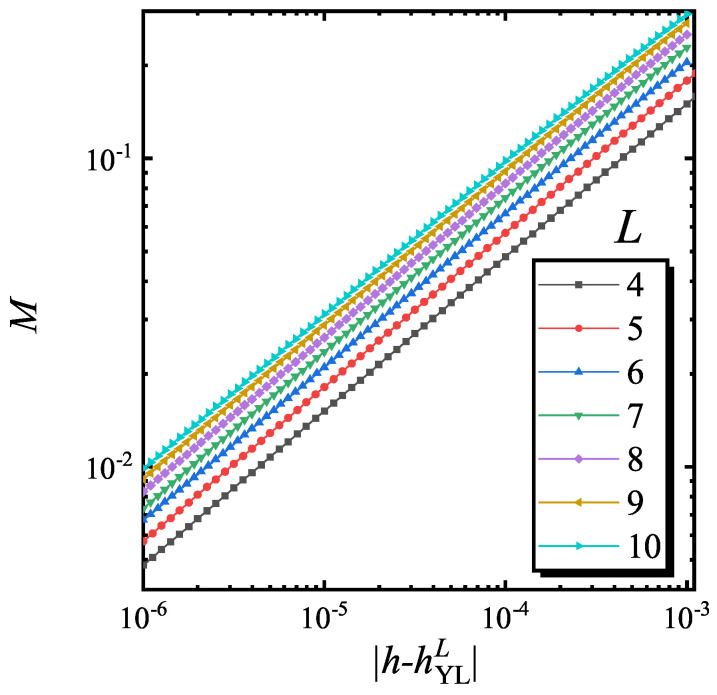
The curves of *M* versus $h-h_{\mathrm{YL}}^{L}$ for different lattice size and g=0.01. The power-law fitting gives that $\sigma_{0}=0.4997\pm 0.0005$.

Firstly, the properties of *M* in the (0+1) D YLES critical region is studied. It is found that at a fixed *g*, static *M* satisfies

(6) $$\begin{array}{c} M\propto |h-h_{\mathrm{YL}}^{L}{|}^{\sigma_{0}}, \end{array}$$

where $\sigma_{0}>0$ is the (0+1) D YLES critical exponent for *M*. The positive $\sigma_{0}$ is different from the divergent behaviors of usual parameters. In Figure 1, *M* versus $|h-h_{\mathrm{YL}}^{L}|$ for different lattice size with g=0.01 are plotted. In the double logarithmic coordinates, these curves are parallel straight lines. The power law fitting results show that $\sigma_{0}=0.4997\pm 0.0005$. Furthermore, it is interesting that *M* is nearly the inverse of $M_{R}$ around the YLES point, since $M_{R}\propto {\left|h-h_{\mathrm{YL}}^{L}\right|}^{-0.5}$ [34]. Therefore, it is expected that one can experimentally measure *M* ($M_{R}$) by measuring $M_{R}$ (*M*) around YLES.

Next, the driven dynamics of *M* is studied by changing *h* for a fixed *g*. Here, we study the linear driving, namely *h* changes in the way of $h=h_{0}+Rt$ across the critical region, since this kind of driving is widely used and readily realized in experiments. *R* is the varying rate of *h*, and it is chosen small enough and $h_{0}$ is irrelevant.

For the (0+1) D YLES critical region, it has been demonstrated that the KZS mechanism was still applicable to the driven dynamics [34]. Since the YLES can appear at finite lattice size, the driven dynamics is characterized by *h* and *R* under fixed *L*. The scaling function satisfies [34,47,48]

(7) $$\begin{array}{c} M\left(h-h_{\mathrm{YL}}^{L},R\right)=R^{s_{0}}f_{1}\left[\left(h-h_{\mathrm{YL}}^{L}\right)R^{\frac{\beta_{0}\delta_{0}}{\nu_{0}r_{0}}}\right]. \end{array}$$

In Equation (7), $s_{0}$ is the scaling factor of *M* for the driven dynamics, and $r_{0}=z_{0}+\beta_{0}\delta_{0}/\nu_{0}$ with $z_{0}$, $\beta_{0}$ and $\nu_{0}$ being the usual critical exponents for the (0+1) D YLES as shown in Table 1 [25,34], and $f_{1}$ is the scaling function of the (0+1) D YLES driven dynamics. It should be noted that the lattice size is not involved in this scaling function. At $h_{\mathrm{YL}}^{L}$, $M_{\mathrm{YL}}^{L}=M\left(h_{\mathrm{YL}}^{L}-h_{\mathrm{YL}}^{L},R\right)\propto R^{s_{0}}$. Therefore, by fitting the curve of $M_{\mathrm{YL}}^{L}$ verse *R*, $s_{0}$ can be determined.

To verify the scaling function Equation (7), we numerically solved Schrödinger equation for model Equation (1) under periodic boundary condition. The finite difference method in the time direction is used, and the time interval is chosen as $2\times 10^{-5}$ in our calculation. Initial condition being $h=h_{0}$, the exact diagonalization is carried out. The lowest real eigenenergy is obtained and the corresponding right eigenstate is set as the initial state. Since the Hamiltonian is non-Hermitian, the wave function is normalized as $\langle \Psi_{R}^{*}|\Psi_{R}\rangle =1$ after each step of evolution.

**Figure 2:**
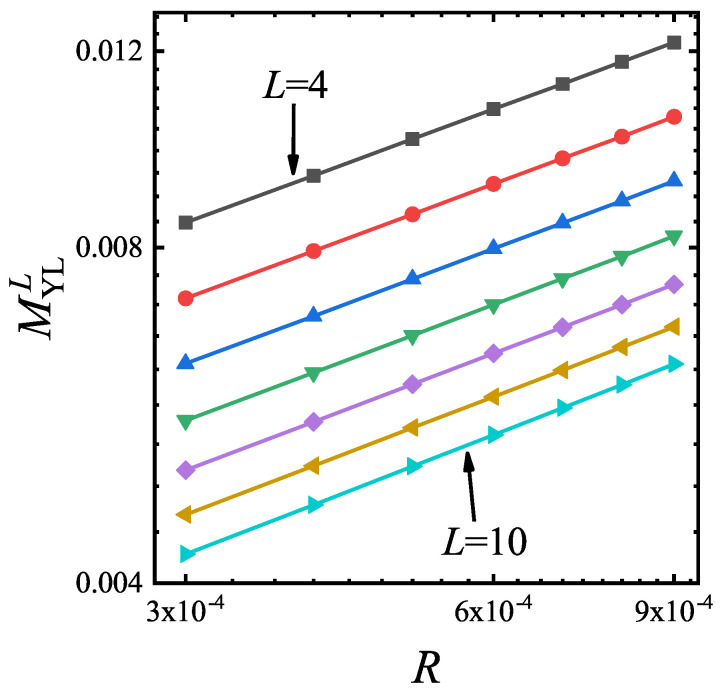
The curves of $M_{\mathrm{YL}}^{L}$ versus *R* for different lattice size and g=3 in the double logarithmic coordinates. Power law fitting gives that $s_{0}=0.3348\pm 0.0007$. From top to bottom, the lattice size is 4 to 10.

**Figure 3:**
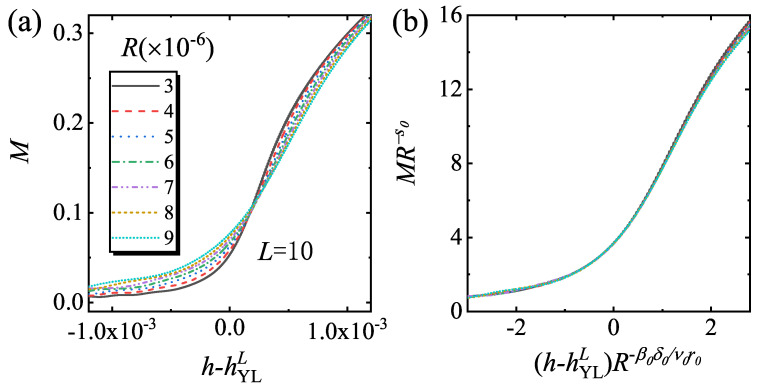
(**a**) The curves of *M* versus $h-h_{\mathrm{YL}}^{L}$ for different *R* with L=10 and (**b**) the rescaled curves of $MR^{-s_{0}}$ versus $\left(h-h_{\mathrm{YL}}^{L}\right)R^{-\beta_{0}\delta_{0}/\nu_{0}r_{0}}$ according to Equation (7). Here, g=0.001 and $h_{\mathrm{YL}}^{L}=0.010558$.

In Figure 2, $M_{\mathrm{YL}}^{L}$ verse *R* for L=4 to 10 are plotted. These lattice sizes correspond to the small and middle size systems. The curves are parallel straight lines in the double logarithmic coordinates, indicating that the scaling function Equation (7) and $s_{0}$ are universal for different size. By a power law fitting, it is found that $s_{0}=0.3348\pm 0.0007$. Since the driven dynamics of $M_{R}$ shows that $M_{R}\left[\left(h_{\mathrm{YL}}^{L}-h_{\mathrm{YL}}^{L},R\right)\right]\propto R^{\beta_{0}/\nu_{0}r_{0}}=R^{-1/3}$[34], $M_{\mathrm{YL}}^{L}$ is also nearly the inverse of $M_{R}\left[\left(h_{\mathrm{YL}}^{L}-h_{\mathrm{YL}}^{L},R\right)\right]$. We select L=10 and calculate the driven dynamics with a fixed *g* to verify the scaling function of Equation (7). In Figure 3, *M* versus *h* for different *R* and the rescaled curves are plotted. The rescaled curves collapse onto each other, indicating that the driven dynamics can be well described by scaling function Equation (7).

### 3.2. Critical Behaviors of *M* in the (1+1) D FPPT and (1+1) D YLES Critical Region

In this subsection, the critical behaviors of *M* in the critical regions of (1+1) D FPPT and (1+1) D YLES is studied.

For the (1+1) D FPPT critical region, the order parameter *M* should satisfy a similar scaling form in the real longitudinal-field case. The static *M* satisfies the following relation [35,47].

(8) $$\begin{array}{c} M=L^{-\beta_{1}/\nu_{1}}f_{2}\left(gL^{1/\nu_{1}},hL^{\beta_{1}\delta_{1}/\nu_{1}}\right). \end{array}$$

Here, $f_{2}$ is the scaling function for *M*, and $\beta_{1}$, $\delta_{1}$ and $\nu_{1}$ are the 2 D classical FPPT critical exponents as shown in Table 1 [1]. These parameters are used to describe the scaling behaviors of quantum Ising chains.

**Figure 4:**
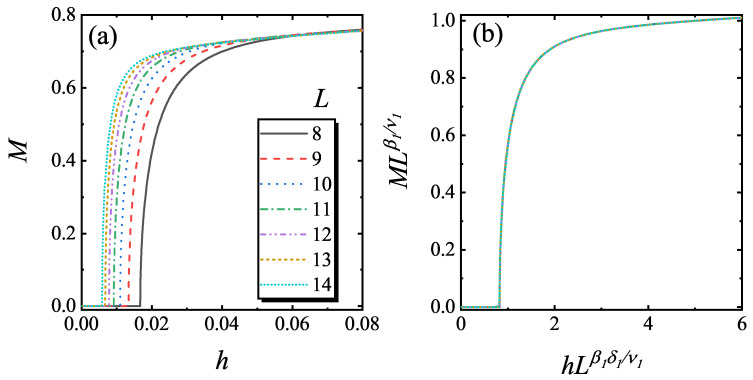
The curves of static *M* versus *h* for different *L* for foxed $gL^{1/\nu_{1}}=0.06$ (**a**) and the rescaled curves of $ML^{\beta_{1}/\nu_{1}}$ versus $hL^{\beta_{1}\delta_{1}/\nu_{1}}$ (**b**) according to Equation (8).

To confirm the relation in Equation (8), the static *M* versus *h* was calculated with a fixed $gL^{1/\nu_{1}}$. Here, we choose that *g* is small and *L* is large, so that the system is in the (1+1) D FPPT critical region. In Figure 4a, *M* versus *h* with different lattice size is plotted. In these curves, the knee points correspond to YLES points $h_{\mathrm{YL}}^{L}$. After rescaling according to Equation (8), the rescaled curves of $ML^{\beta_{1}/\nu_{1}}$ versus $hL^{\beta_{1}\delta_{1}/\nu_{1}}$ match with each other as shown in Figure 4b.

**Figure 5:**
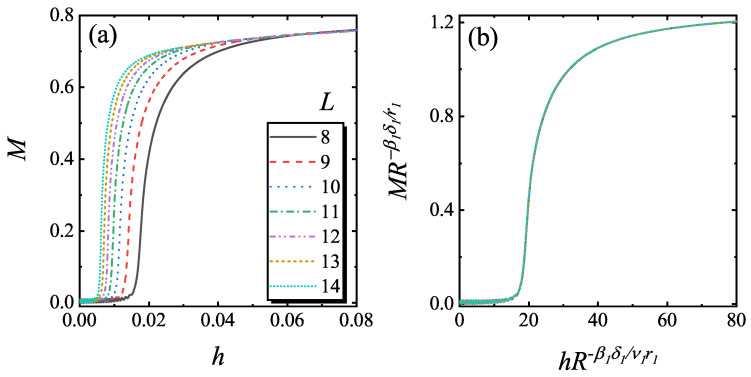
(**a**) The curves of *M* versus *h* for different *L* with $gR^{-\frac{1}{\nu r}}=0.3133$ and $L^{-1}R^{1\frac{1}{r}}=0.1915$ and (**b**) the rescaled curves of $MR^{-\beta \delta /r}$ versus $hR^{-\beta \delta /\nu r}$ according to Equation (9).

The driven dynamics in the (1+1) D FPPT critical region is characterized by *h*, *R* and *L*. The scaling form of *M* reads [35]

(9) $$\begin{array}{c} M\left(g,h,L,R\right)=R^{\frac{\beta_{1}}{\nu_{1}r_{1}}}f_{3}\left(gR^{-\frac{1}{\nu_{1}r_{1}}},L^{-1}R^{-\frac{1}{r_{1}}},hR^{-\frac{\beta_{1}\delta_{1}}{\nu_{1}r_{1}}}\right), \end{array}$$

where $r_{1}$ and $z_{1}$ are the critical exponent of the (1+1) D FPPT and $f_{3}$ is a scaling function. By fixing $gR^{-\frac{1}{\nu_{1}r_{1}}}$ and $L^{-1}R^{-\frac{1}{r_{1}}}$ in Equation (9), the curves of *M* as a function of *h* for different lattice size are calculated. The results are plotted in Figure 5a. The rescaled curves of $MR^{-\frac{\beta_{1}}{\nu_{1}r_{1}}}$ versus $hR^{-\frac{\beta_{1}\delta_{1}}{\nu_{1}r_{1}}}$ match with each other very well as shown in Figure 5b, which confirms the scaling function of Equation (9). Moreover, from Figure 5a, *M* for different *L* reach a saturation value at large *h* but still less than 1, which means *M* is bounded in the range of [−1,1] as the order parameter defined in the Hermitian system.

Near $h_{\mathrm{YL}}^{\infty }$ for a fixed *g*, the scaling dynamics of *M* of the medium lattice size should satisfy the (1+1) D KZS with the finite-size corrections being considered. Following the (1+1) D YLES scaling theory, the scaling form of *M* reads [34]

(10) $$\begin{array}{c} M\left(h-h_{\mathrm{YL}}^{\infty },R,L\right)=R^{s_{2}}f_{4}\left[\left(h-h_{\mathrm{YL}}^{\infty }\right)R^{-\frac{\beta_{2}\delta_{2}}{\nu_{2}r_{2}}},L^{-1}R^{-1/r_{2}}\right], \end{array}$$

where $\beta_{2}$, $\delta_{2}$, $\nu_{2}$ and $r_{2}$ are the critical exponents of the (1+1) D YLES critical theory (see Table 1) [34], and $s_{2}$ is the (1+1) D YLES critical exponent for *M*. By fixing $L^{-1}R^{-1/r_{2}}$ in Equation (10), $s_{2}$ can be obtained by fitting $M_{\mathrm{YL}}^{\infty }=M\left(h_{\mathrm{YL}}^{\infty }-h_{\mathrm{YL}}^{\infty },R,L\right)$ verse *R*.

**Figure 6:**
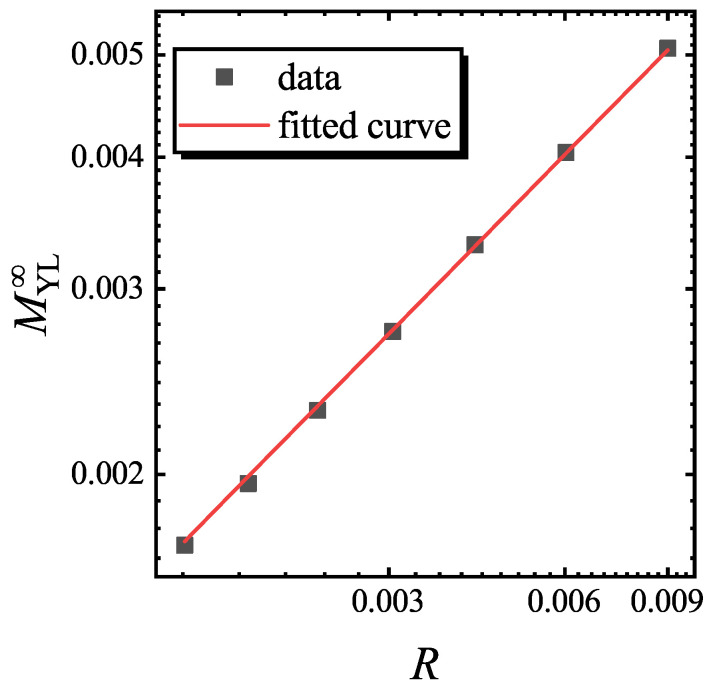
The curve of $M_{\mathrm{YL}}^{\infty }$ versus *R* and the fitted curve with fixed $L^{-1}R^{-1/r_{2}}=2.001689$ in the double logarithmic coordinates. The fitting curve is $M_{\mathrm{YL}}^{\infty }\propto R^{0.5816}$. Here, $h_{\mathrm{YL}}^{\infty }=2.292425$ and g=4.

**Figure 7:**
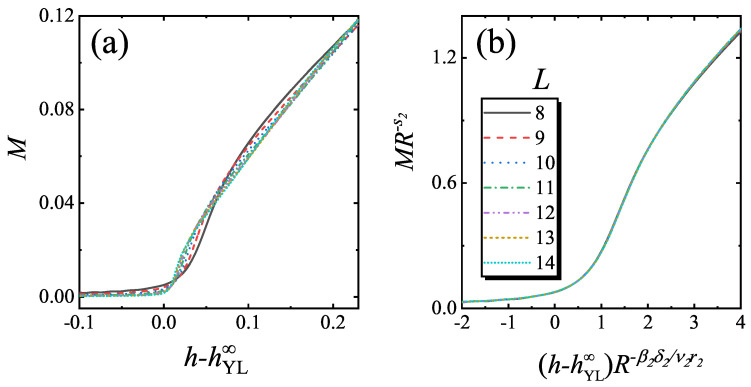
(**a**) The curves of *M* versus *h* for different *L* with $L^{-1}R^{-1/r_{2}}=2.001689$ and (**b**) the rescaled curves of $MR^{-s_{2}}$ versus $\left(h-h_{\mathrm{YL}}^{\infty }\right)R^{-\beta_{2}\delta_{2}/\nu_{2}r_{2}}$ according to Equation (10). Here, $h_{\mathrm{YL}}^{\infty }=2.292425$ and g=4.

To verify the scaling function of Equation (10), $s_{2}$ is firstly determined by fitting $M_{\mathrm{YL}}^{\infty }$ versus *R* for a fixed $L^{-1}R^{-1/r_{2}}$. Here, *g* is chosen as g=4, which is large enough to ensure that the system is not in the critical region of (1+1) D YLES. In Figure 6, $M_{\mathrm{YL}}^{\infty }$ versus *R* and the fitted curve are plotted, and it is achieved that $s_{2}=0.5816$. In Figure 7, *M* verses *h* and the rescaled curves according to Equation (10) are plotted. We find that the rescaled curve matches with each other, confirming Equation (10).

Finally, we have some discussion with respect the defined *M*. Suppose that the wave functions have been normalization, $\langle \psi_{n}^{L}|\psi_{n}^{R}\rangle =1$. Then

(11) $$\begin{array}{ccc} M_{Q} & = & \frac{1}{\langle \psi_{g}^{R*}|\psi_{g}^{R}\rangle }\langle \psi_{g}^{R*}|\psi_{g}^{R}\rangle \langle \psi_{g}^{L}|\hat{M}|\psi_{g}^{R}\rangle . \end{array}$$

Now we use the complete set Equation (4), so as to get $|\psi_{g}^{R}\rangle \langle \psi_{g}^{L}|=1-\sum_{nog}|\psi_{g}^{R}\rangle \langle \psi_{g}^{L}|$. One immediately find that

(12) $$\begin{array}{c} M_{Q}=M-\frac{1}{\langle \psi_{g}^{R*}|\psi_{g}^{R}\rangle }\langle \psi_{g}^{R*}|\sum_{no\;g}|\psi_{n}^{R}\rangle \langle \psi_{n}^{L}|\hat{M}|\psi_{g}^{R}\rangle , \end{array}$$

which means that the behavior of $M_{Q}$ includes *M* and the information of the highly excited states. Moreover, We have confirmed numerically that the static behavior of the second term in Equation (12) have similar scaling behaviors but opposite with $M_{Q}$ around the YLES points. Therefore, the divergence of $M_{Q}$ and the second term in Equation (12) can result in a non-divergence behavior of *M*. From this equation it is understood that the divergence is from the contribution of excited states. The ground state itself does not yield divergence. The behavior of $M_{Q}$ reflects the effect from all possible excited states, while the definition of *M* seems get rid of the influence from excited states. Therefore, the definition of *M* is really a good way to present an index which is finite at critical point.

## 4. Summary

In summary, the pseudo-YLES critical behavior has been studied in this paper. An order parameter has been suggested to study the static properties and driven dynamics of pseudo-YLES. We have found that *M* scales as $M\propto |h-h_{\mathrm{YL}}^{L}{|}^{\sigma_{0}}$ around the YLES points, with positive $\sigma_{0}$ that is different from the usual order parameters, and *M* is not divergence at the YLES points. The static and driven dynamics of *M* are studied in the (0+1) D YLES, (1+1) D FPPT and (1+1) D YLES critical regions, and it is found that the scaling behaviors can be described by the (0+1) D YLES, (1+1) D FPPT and (1+1) D YLES scaling theories, respectively. These results demonstrate that *M* could be a good indicator of phase transition in YLES. Moreover, since the static *M* is finite around the YLES points, it is expected that *M* may be easier to measure experimentally rather than the usual order parameters. Although not shown here, the order parameter can also be defined in the left eigenvector space, and the order parameter in the left eigenvector space also satisfies the conclusions obtained above.

## Figures and Tables

**Table 1:** Critical exponents for the (0+1) D YLES, (1+1) D FPPT and (1+1) D YLES, respectively.

(0+1) D YLES	$\nu_{0}$	$\beta_{0}$	$\delta_{0}$	$z_{0}$	$r_{0}$
	−1	1	−2	1	3
(1+1) D FPPT	$\nu_{1}$	$\beta_{1}$	$\delta_{1}$	$z_{1}$	$r_{1}$
	1	1/8	15	1	23/8
(1+1) D YLES	$\nu_{2}$	$\beta_{2}$	$\delta_{2}$	$z_{2}$	$r_{2}$
	−5/2	1	−6	1	3.4

## Abbreviations

The following abbreviations are used in this manuscript:

YLES	Yang-Lee edge singularity
EP	exceptional point
FPPT	ferromagnetic-paramagnetic phase transition
KZS	Kibble-Zurek scaling
